# Supplementary material for: Smooth Interpolating Curves with Local Control and Monotone Alternating Curvature
Source: Comput Graph Forum. 2022 Oct 6;41(5):25–38. doi: 10.1111/cgf.14600 (PMC9827861; doi:10.1111/cgf.14600)
Supplement: Supplementary file 1 — Supplement Material [file CGF-41-25-s001.zip › Local-Smooth-Interpolating-MonoCurvature/extern/clothoids/submodules/Utils/docs/api-cpp/program_listing_file_os.cc.html]

Program Listing for File os.cc — Utils v1.2

### Navigation

- index
- toc
- Utils »
- Program Listing for File os.cc

# Program Listing for File os.cc¶

↰ Return to documentation for file (`os.cc`)

```
// Formatting library for C++ - optional OS-specific functionality
//
// Copyright (c) 2012 - 2016, Victor Zverovich
// All rights reserved.
//
// For the license information refer to format.h.

// Disable bogus MSVC warnings.
#if !defined(_CRT_SECURE_NO_WARNINGS) && defined(_MSC_VER)
#  define _CRT_SECURE_NO_WARNINGS
#endif

#include "fmt/os.h"

#include <climits>

#if FMT_USE_FCNTL
#  include <sys/stat.h>
#  include <sys/types.h>

#  ifndef _WIN32
#    include <unistd.h>
#  else
#    ifndef WIN32_LEAN_AND_MEAN
#      define WIN32_LEAN_AND_MEAN
#    endif
#    include <io.h>

#    ifndef S_IRUSR
#      define S_IRUSR _S_IREAD
#    endif
#    ifndef S_IWUSR
#      define S_IWUSR _S_IWRITE
#    endif
#    ifndef S_IRGRP
#      define S_IRGRP 0
#    endif
#    ifndef S_IROTH
#      define S_IROTH 0
#    endif
#  endif  // _WIN32
#endif    // FMT_USE_FCNTL

#ifdef _WIN32
#  include <windows.h>
#endif

#ifdef fileno
#  undef fileno
#endif

namespace {
#ifdef _WIN32
// Return type of read and write functions.
using rwresult = int;

// On Windows the count argument to read and write is unsigned, so convert
// it from size_t preventing integer overflow.
inline unsigned convert_rwcount(std::size_t count) {
  return count <= UINT_MAX ? static_cast<unsigned>(count) : UINT_MAX;
}
#elif FMT_USE_FCNTL
// Return type of read and write functions.
using rwresult = ssize_t;

inline std::size_t convert_rwcount(std::size_t count) { return count; }
#endif
}  // namespace

FMT_BEGIN_NAMESPACE

#ifdef _WIN32
detail::utf16_to_utf8::utf16_to_utf8(basic_string_view<wchar_t> s) {
  if (int error_code = convert(s)) {
    FMT_THROW(windows_error(error_code,
                            "cannot convert string from UTF-16 to UTF-8"));
  }
}

int detail::utf16_to_utf8::convert(basic_string_view<wchar_t> s) {
  if (s.size() > INT_MAX) return ERROR_INVALID_PARAMETER;
  int s_size = static_cast<int>(s.size());
  if (s_size == 0) {
    // WideCharToMultiByte does not support zero length, handle separately.
    buffer_.resize(1);
    buffer_[0] = 0;
    return 0;
  }

  int length = WideCharToMultiByte(CP_UTF8, 0, s.data(), s_size, nullptr, 0,
                                   nullptr, nullptr);
  if (length == 0) return GetLastError();
  buffer_.resize(length + 1);
  length = WideCharToMultiByte(CP_UTF8, 0, s.data(), s_size, &buffer_[0],
                               length, nullptr, nullptr);
  if (length == 0) return GetLastError();
  buffer_[length] = 0;
  return 0;
}

namespace detail {

class system_message {
  system_message(const system_message&) = delete;
  void operator=(const system_message&) = delete;

  unsigned long result_;
  wchar_t* message_;

  static bool is_whitespace(wchar_t c) FMT_NOEXCEPT {
    return c == L' ' || c == L'\n' || c == L'\r' || c == L'\t' || c == L'\0';
  }

 public:
  explicit system_message(unsigned long error_code)
      : result_(0), message_(nullptr) {
    result_ = FormatMessageW(
        FORMAT_MESSAGE_ALLOCATE_BUFFER | FORMAT_MESSAGE_FROM_SYSTEM |
            FORMAT_MESSAGE_IGNORE_INSERTS,
        nullptr, error_code, MAKELANGID(LANG_NEUTRAL, SUBLANG_DEFAULT),
        reinterpret_cast<wchar_t*>(&message_), 0, nullptr);
    if (result_ != 0) {
      while (result_ != 0 && is_whitespace(message_[result_ - 1])) {
        --result_;
      }
    }
  }
  ~system_message() { LocalFree(message_); }
  explicit operator bool() const FMT_NOEXCEPT { return result_ != 0; }
  operator basic_string_view<wchar_t>() const FMT_NOEXCEPT {
    return basic_string_view<wchar_t>(message_, result_);
  }
};

class utf8_system_category final : public std::error_category {
 public:
  const char* name() const FMT_NOEXCEPT override { return "system"; }
  std::string message(int error_code) const override {
    system_message msg(error_code);
    if (msg) {
      utf16_to_utf8 utf8_message;
      if (utf8_message.convert(msg) == ERROR_SUCCESS) {
        return utf8_message.str();
      }
    }
    return "unknown error";
  }
};

}  // namespace detail

FMT_API const std::error_category& system_category() FMT_NOEXCEPT {
  static const detail::utf8_system_category category;
  return category;
}

std::system_error vwindows_error(int err_code, string_view format_str,
                                 format_args args) {
  auto ec = std::error_code(err_code, system_category());
  return std::system_error(ec, vformat(format_str, args));
}

void detail::format_windows_error(detail::buffer<char>& out, int error_code,
                                  const char* message) FMT_NOEXCEPT {
  FMT_TRY {
    system_message msg(error_code);
    if (msg) {
      utf16_to_utf8 utf8_message;
      if (utf8_message.convert(msg) == ERROR_SUCCESS) {
        format_to(buffer_appender<char>(out), "{}: {}", message, utf8_message);
        return;
      }
    }
  }
  FMT_CATCH(...) {}
  format_error_code(out, error_code, message);
}

void report_windows_error(int error_code, const char* message) FMT_NOEXCEPT {
  report_error(detail::format_windows_error, error_code, message);
}
#endif  // _WIN32

buffered_file::~buffered_file() FMT_NOEXCEPT {
  if (file_ && FMT_SYSTEM(fclose(file_)) != 0)
    report_system_error(errno, "cannot close file");
}

buffered_file::buffered_file(cstring_view filename, cstring_view mode) {
  FMT_RETRY_VAL(file_, FMT_SYSTEM(fopen(filename.c_str(), mode.c_str())),
                nullptr);
  if (!file_)
    FMT_THROW(system_error(errno, "cannot open file {}", filename.c_str()));
}

void buffered_file::close() {
  if (!file_) return;
  int result = FMT_SYSTEM(fclose(file_));
  file_ = nullptr;
  if (result != 0) FMT_THROW(system_error(errno, "cannot close file"));
}

// A macro used to prevent expansion of fileno on broken versions of MinGW.
#define FMT_ARGS

int buffered_file::fileno() const {
  int fd = FMT_POSIX_CALL(fileno FMT_ARGS(file_));
  if (fd == -1) FMT_THROW(system_error(errno, "cannot get file descriptor"));
  return fd;
}

#if FMT_USE_FCNTL
file::file(cstring_view path, int oflag) {
#  ifdef _WIN32
  using mode_t = int;
#  endif
  mode_t mode = S_IRUSR | S_IWUSR | S_IRGRP | S_IROTH;
#  if defined(_WIN32) && !defined(__MINGW32__)
  fd_ = -1;
  FMT_POSIX_CALL(sopen_s(&fd_, path.c_str(), oflag, _SH_DENYNO, mode));
#  else
  FMT_RETRY(fd_, FMT_POSIX_CALL(open(path.c_str(), oflag, mode)));
#  endif
  if (fd_ == -1)
    FMT_THROW(system_error(errno, "cannot open file {}", path.c_str()));
}

file::~file() FMT_NOEXCEPT {
  // Don't retry close in case of EINTR!
  // See http://linux.derkeiler.com/Mailing-Lists/Kernel/2005-09/3000.html
  if (fd_ != -1 && FMT_POSIX_CALL(close(fd_)) != 0)
    report_system_error(errno, "cannot close file");
}

void file::close() {
  if (fd_ == -1) return;
  // Don't retry close in case of EINTR!
  // See http://linux.derkeiler.com/Mailing-Lists/Kernel/2005-09/3000.html
  int result = FMT_POSIX_CALL(close(fd_));
  fd_ = -1;
  if (result != 0) FMT_THROW(system_error(errno, "cannot close file"));
}

long long file::size() const {
#  ifdef _WIN32
  // Use GetFileSize instead of GetFileSizeEx for the case when _WIN32_WINNT
  // is less than 0x0500 as is the case with some default MinGW builds.
  // Both functions support large file sizes.
  DWORD size_upper = 0;
  HANDLE handle = reinterpret_cast<HANDLE>(_get_osfhandle(fd_));
  DWORD size_lower = FMT_SYSTEM(GetFileSize(handle, &size_upper));
  if (size_lower == INVALID_FILE_SIZE) {
    DWORD error = GetLastError();
    if (error != NO_ERROR)
      FMT_THROW(windows_error(GetLastError(), "cannot get file size"));
  }
  unsigned long long long_size = size_upper;
  return (long_size << sizeof(DWORD) * CHAR_BIT) | size_lower;
#  else
  using Stat = struct stat;
  Stat file_stat = Stat();
  if (FMT_POSIX_CALL(fstat(fd_, &file_stat)) == -1)
    FMT_THROW(system_error(errno, "cannot get file attributes"));
  static_assert(sizeof(long long) >= sizeof(file_stat.st_size),
                "return type of file::size is not large enough");
  return file_stat.st_size;
#  endif
}

std::size_t file::read(void* buffer, std::size_t count) {
  rwresult result = 0;
  FMT_RETRY(result, FMT_POSIX_CALL(read(fd_, buffer, convert_rwcount(count))));
  if (result < 0) FMT_THROW(system_error(errno, "cannot read from file"));
  return detail::to_unsigned(result);
}

std::size_t file::write(const void* buffer, std::size_t count) {
  rwresult result = 0;
  FMT_RETRY(result, FMT_POSIX_CALL(write(fd_, buffer, convert_rwcount(count))));
  if (result < 0) FMT_THROW(system_error(errno, "cannot write to file"));
  return detail::to_unsigned(result);
}

file file::dup(int fd) {
  // Don't retry as dup doesn't return EINTR.
  // http://pubs.opengroup.org/onlinepubs/009695399/functions/dup.html
  int new_fd = FMT_POSIX_CALL(dup(fd));
  if (new_fd == -1)
    FMT_THROW(system_error(errno, "cannot duplicate file descriptor {}", fd));
  return file(new_fd);
}

void file::dup2(int fd) {
  int result = 0;
  FMT_RETRY(result, FMT_POSIX_CALL(dup2(fd_, fd)));
  if (result == -1) {
    FMT_THROW(system_error(errno, "cannot duplicate file descriptor {} to {}",
                           fd_, fd));
  }
}

void file::dup2(int fd, std::error_code& ec) FMT_NOEXCEPT {
  int result = 0;
  FMT_RETRY(result, FMT_POSIX_CALL(dup2(fd_, fd)));
  if (result == -1) ec = std::error_code(errno, std::generic_category());
}

void file::pipe(file& read_end, file& write_end) {
  // Close the descriptors first to make sure that assignments don't throw
  // and there are no leaks.
  read_end.close();
  write_end.close();
  int fds[2] = {};
#  ifdef _WIN32
  // Make the default pipe capacity same as on Linux 2.6.11+.
  enum { DEFAULT_CAPACITY = 65536 };
  int result = FMT_POSIX_CALL(pipe(fds, DEFAULT_CAPACITY, _O_BINARY));
#  else
  // Don't retry as the pipe function doesn't return EINTR.
  // http://pubs.opengroup.org/onlinepubs/009696799/functions/pipe.html
  int result = FMT_POSIX_CALL(pipe(fds));
#  endif
  if (result != 0) FMT_THROW(system_error(errno, "cannot create pipe"));
  // The following assignments don't throw because read_fd and write_fd
  // are closed.
  read_end = file(fds[0]);
  write_end = file(fds[1]);
}

buffered_file file::fdopen(const char* mode) {
// Don't retry as fdopen doesn't return EINTR.
#  if defined(__MINGW32__) && defined(_POSIX_)
  FILE* f = ::fdopen(fd_, mode);
#  else
  FILE* f = FMT_POSIX_CALL(fdopen(fd_, mode));
#  endif
  if (!f)
    FMT_THROW(
        system_error(errno, "cannot associate stream with file descriptor"));
  buffered_file bf(f);
  fd_ = -1;
  return bf;
}

long getpagesize() {
#  ifdef _WIN32
  SYSTEM_INFO si;
  GetSystemInfo(&si);
  return si.dwPageSize;
#  else
  long size = FMT_POSIX_CALL(sysconf(_SC_PAGESIZE));
  if (size < 0) FMT_THROW(system_error(errno, "cannot get memory page size"));
  return size;
#  endif
}

FMT_API void ostream::grow(size_t) {
  if (this->size() == this->capacity()) flush();
}
#endif  // FMT_USE_FCNTL
FMT_END_NAMESPACE
```

### Quick search

### Table of Contents

- rang - Colors for your Terminal
- Zstr
- C++ API

«
hide menu

menu
sidebar
»

### Navigation

- index
- toc
- Utils »
- Program Listing for File os.cc

© Copyright 2021, Enrico Bertolazzi.
Created using Sphinx 4.2.0.
